# A Blended Intervention Targeting Emotion Dysregulation in Adults With Attention-Deficit/Hyperactivity Disorder: Development and Feasibility Study

**DOI:** 10.2196/53931

**Published:** 2024-01-17

**Authors:** Emilie S Nordby, Frode Guribye, Viktor Schønning, Sander Lindholm Andersen, Jonna Kuntsi, Astri J Lundervold

**Affiliations:** 1 Division of Psychiatry Haukeland University Hospital Bergen Norway; 2 Department of Biological and Medical Psychology Faculty of Psychology University of Bergen Bergen Norway; 3 Department of Information Science and Media Studies Faculty of Social Sciences University of Bergen Bergen Norway; 4 Department of Child and Adolescent Psychiatry Division of Psychiatry Haukeland University Hospital Bergen Norway; 5 Social, Genetic and Developmental Psychiatry Centre Institute of Psychiatry, Psychology and Neuroscience King’s College London London United Kingdom

**Keywords:** ADHD, adult, adults, app, applications, apps, attention deficit, blended intervention, blended, develop, development, digital, emotion regulation, emotion, emotional, emotions, feasibility, group session, group sessions, hybrid, hyperactivity, inattention, mental health, neurodevelopmental, psychotherapy, satisfaction, skill, training

## Abstract

**Background:**

Many adults with attention-deficit/hyperactivity disorder (ADHD) experience difficulties related to emotion regulation. Such difficulties are known to substantially impact quality of life and overall functioning. Yet, there is a lack of treatment interventions specifically designed to address these challenges.

**Objective:**

This study aimed to describe the development and assess the feasibility, along with the initial clinical outcomes, of a novel blended intervention for adults with ADHD. The blended intervention combines both face-to-face and digital components and is specifically designed to address emotion dysregulation in ADHD.

**Methods:**

This intervention was an 8-week blended intervention combining weekly face-to-face group sessions with a supplementary digital companion app. The intervention is based on elements from dialectic behavioral therapy skills training and positive psychology. To evaluate its feasibility, we performed a 10-week feasibility study with an uncontrolled pre-post study design, including 16 adults with ADHD and co-occurring emotion dysregulation. The feasibility measures encompassed adherence, satisfaction, and perceived credibility of the intervention. Clinical outcomes were evaluated by self-reported symptoms of emotion dysregulation, inattention, hyperactivity-impulsivity, executive function, depression, anxiety, and a measure of quality of life. Paired sample 2-tailed *t* tests were used to analyze clinical outcomes with a Bonferroni-corrected significance level.

**Results:**

Both treatment credibility and treatment satisfaction were rated favorably by the majority of the participants. In particular, the participants emphasized meeting others with ADHD as beneficial. In terms of adherence, 3 participants withdrew before initiating the intervention, while another 4 participants did not complete the intervention. On average, the participants who enrolled in the intervention attended 6.2 of the 8 group sessions and completed 6.7 of the 8 skills training modules in the companion app. In terms of clinical outcomes, there was a reduction in symptoms of emotion dysregulation from before to after the intervention (*d*=2.0). Significant improvements were also observed in measures of inattention (*d*=1.1) and hyperactivity-impulsivity (*d*=0.9). However, no significant improvements were found in the domains of depression, anxiety, quality of life, and executive functioning.

**Conclusions:**

The results are encouraging, both in terms of feasibility and the preliminary clinical results on emotion dysregulation. The blended format, combining digital and face-to-face elements, may also seem to offer some advantages: the group-based format was valued as it facilitated peer interaction, while a rather high completion of modules in the companion app highlights its potential to enhance skills training between the group sessions. Future randomized controlled trials are called for to further evaluate the clinical effectiveness of the intervention.

**Trial Registration:**

ClinicalTrials.gov NCT05644028; https://clinicaltrials.gov/study/NCT05644028

## Introduction

Attention-deficit/hyperactivity disorder (ADHD) affects approximately 2.6% of the adult population [1]. Although symptoms of ADHD initially present themselves in childhood, most individuals diagnosed with ADHD as children continue to experience symptoms and associated impairments into adulthood [2]. Moreover, there is a rising trend of individuals first receiving their ADHD diagnosis in adulthood [3]. ADHD manifests through symptoms of inattention, hyperactivity, and impulsivity that significantly disrupt the individual’s daily functioning [4]. Beyond these core symptoms, ADHD is accompanied by an array of co-occurring symptoms and difficulties, both somatic and psychological, which can further complicate and amplify the challenges associated with the diagnosis [5].

Emotion dysregulation is a common deficit observed across many mental health conditions [6]. It is characterized by challenges in effectively managing and modulating one’s emotions, including the emotional experience (eg, intensity and duration) and expression [7]. Emotion dysregulation commonly coexists with ADHD, impacting as many as 34%-70% of the adults with the diagnosis [8]. Emotion dysregulation also occurs in adults with ADHD without the presence of other comorbid diagnoses [9], giving support to the notion that it is a core component of ADHD [10-12]. The co-occurrence of emotion dysregulation with ADHD is linked to a range of negative outcomes, including occupational challenges, interpersonal conflicts, financial struggles, parenting stress, as well as tendencies for self-harm, illicit drug use, and suicidal ideation [13-15]. Moreover, research indicates that emotion dysregulation serves as an independent predictor of impairment in ADHD [16]. Its adverse influence on self-esteem and quality of life has further been observed to exceed the effects of inattention and hyperactivity [17]. Given the high prevalence of emotion dysregulation in ADHD and the associated negative outcomes, adults with ADHD should be offered treatment interventions that specifically aim to strengthen their emotion regulation skills.

While pharmacological treatment remains the main treatment approach for the management of ADHD in adults, the UK National Institute for Health and Care Excellence recommends supplementing with psychological interventions, such as psychoeducation or psychotherapy [18]. Psychological alternatives are especially critical in cases where the individual does not want to take medications or when medications either do not lead to sufficient clinical improvement or result in unwanted side effects. This may be particularly relevant for those with co-occurring emotion dysregulation, as ADHD medications appear to be less effective for these difficulties [19]. A systematic review and meta-analysis showed that traditional ADHD medications, including methylphenidate, atomoxetine, and lisdexamfetamine, only had small to moderate effects on emotion dysregulation among adults, which were significantly lower than the effect sizes reported for core symptoms of inattention, hyperactivity, and impulsivity [20]. As such, the authors of the abovementioned review emphasize that there is a need for more research on both pharmacological and psychological treatments targeting emotion dysregulation in adults with ADHD [20].

There is limited access to psychological treatments among adults with ADHD, and most available interventions mainly address the core symptoms of inattention and hyperactivity [21,22]. To the authors’ knowledge, there are currently 8 studies that have included emotion dysregulation as an outcome measure in studies of psychological interventions for adults with ADHD, including 2 randomized controlled trials [23-30]. However, only 2 of the studies reported emotion dysregulation to be the primary treatment target [24,26]. Carroll et al [26] developed and tested the psychological intervention “Group Therapy for Improving Emotional Acceptance and Regulatory Skills in Adults with ADHD” (GEARS), which consists of 14 weekly group sessions. In an uncontrolled pilot study with 226 participants, both treatment credibility and treatment satisfaction were rated as high, and preliminary clinical effects showed a reduction in emotion dysregulation symptoms with large effect sizes [26]. These findings are also supported by other studies [23,25,27-29]. On the other hand, Halmøy et al [24] did not find any significant difference in measures of emotion regulation between the control group and the treatment group in a randomized controlled trial of a 14-week dialectic behavioral therapy–based intervention. With the current evidence, it is thus premature to conclude whether psychological interventions are effective for adults with ADHD with co-occurring emotion dysregulation.

Digital psychological interventions have become increasingly popular in the past decade. The inclusion of digital tools in treatment interventions for adults with ADHD may be useful in addressing common challenges such as forgetfulness, nonadherence to treatment, and incomplete homework assignments [31,32]. For example, content from face-to-face therapy sessions, such as coping skills or psychoeducation, could be made available on the web through a website or a mobile app. The increased accessibility of such treatment elements may facilitate the generalization of therapeutic skills in everyday life for the clients, which is a central aim of most psychological interventions [33]. Blended interventions, where elements from face-to-face and digital treatment formats are combined, could be advantageous as they use the strengths of both treatment formats [33]. A systematic review of blended interventions in mental health care suggests that these interventions may save clinician’s time, lead to lower dropout rates, and help to maintain positive changes made in psychotherapy over time [34]. However, there have been few studies examining a blended treatment format among adults with ADHD. To our knowledge, there has been 1 randomized controlled trial examining the effect of a mobile app to deliver psychoeducation in a group-based intervention for adults with ADHD [35]. In this study, it was found that the participants who used the mobile companion app had a greater reduction in ADHD symptoms and a higher completion rate of homework assignments as compared to participants who received a printed version of the psychoeducation [35]. As such, this study provides evidence that psychological interventions for adults with ADHD may be augmented by the implementation of digital tools.

The overall aim of this study was to describe the development of and assess the feasibility and preliminary clinical effects of a blended psychological intervention for adults with ADHD. This intervention integrates face-to-face group sessions with a digital companion app for skills training, designed to address emotion regulation. The developmental process and the core content of the intervention are outlined in the methods section. Through the feasibility study of the intervention, the following research questions were addressed:

1. How do participants with ADHD rate their satisfaction and the credibility of the intervention?
2. What is the participants’ adherence level to the intervention?
3. What are the preliminary clinical effects of the intervention on emotion regulation, inattention, hyperactivity-impulsivity, quality of life, anxiety, depression, and executive functioning?

## Methods

### Phase 1: Development of Intervention

#### Participants

The participants who took part in the development phase of the intervention included 5 adults with ADHD, 2 clinical psychologists, 1 clinical psychology student, 2 human-computer interaction experts, and 2 user experience designers. The adults with ADHD were originally recruited from the local ADHD patient association. The adults with ADHD received a gift certificate worth NOK 400 (US $37) in total for their participation in the design workshop and evaluation meeting.

#### Methods of Development

The intervention was developed over 2 years through an iterative process. Multiple methods were used during this development (Table 1 shows an overview of methods and findings).

**Table 1:** Overview of the methods applied in the development of the intervention and key findings in the 2-year developmental process.

Method	Content	Findings
Synthesis of quantitative literature	A synthesis of previous studies examining psychological interventions targeting emotion dysregulation in ADHD^a^ was conducted. In this step, we examined the format, treatment approach, and common psychotherapeutic elements in the interventions.	Various psychological frameworks have been used in interventions targeting emotion dysregulation in ADHD, including DBT^b^, cognitive behavioral therapy, goal management training, and mentalization-based therapy. A group-based format has been applied in previous interventions. Most previous interventions apply a structured and manualized format. Most previous interventions use homework assignments to generalize skills. Common intervention elements were psychoeducation, mindfulness, acceptance strategies, self-monitoring, emotion regulation skills, behavioral analysis, planning and organization strategies, communication skills, and problem-solving skills.
Synthesis of qualitative literature	A synthesis of previous qualitative examining the experience of participating in psychological intervention for adults with ADHD was conducted. In this step, we sought to understand the needs and preferences of psychological interventions among adults with ADHD.	In previous interventions, the opportunity to share experiences with peers was perceived as valuable by adults with ADHD. Adults with ADHD report that they prefer an emphasis on strengths and solutions in treatment. Incorporation of digital tools in treatment was perceived as useful by adults with ADHD.
Expert meetings	The development process included several expert meetings. In these meetings, the format and elements of the intervention were discussed and reviewed. Suggestions regarding intervention content and design were made based on clinical expertise and experience.	Beneficial elements that should be included in the intervention based on clinical experience: group-based format, focus on positive aspects, option to choose from a variety of coping strategies, interactive in-class exercises and discussions, and short homework assignments. Design considerations and suggestions for the companion app: clear structure, minimizing distraction, clear information, and use of rewards and praise.
Co-design workshop	The workshop included 5 adults with ADHD, 2 clinical psychologists, and 2 HCI^c^ experts. In the workshop, the adults were given information about the project and a general idea of the intervention. Following this, the adults were asked about challenging situations in terms of emotion dysregulation, common coping strategies, and their preferences for intervention content and features.	Emotion dysregulation was a common challenge among adults with ADHD. Perceived useful coping strategies by adults with ADHD: acceptance, self-compassion, distraction, reappraisal, time-out, and relaxation. Useful features in companion app: reminders, note-taking, overview of coping strategies, calendar, peer support, and inclusion of videos.
Design sprint of companion app	A design sprint lasting 5 days was conducted to create a prototype of the companion app. An HCI expert led the design sprint, with the inclusion of 2 UX^d^ designers and 1 clinical psychologist as participants.	A prototype of the companion app was completed, including design and features in the app.
Cocreation of intervention manual	The first author created the intervention manual based on the previous findings. The protocol was then reviewed and revised by 2 other clinical experts, including 1 expert with ADHD.	A first version of the intervention manual was created.
Evaluation seminar with adults with ADHD	The evaluation seminar included the same participants as the co-design workshop. In the first part of the seminar, a clinical psychologist presented the content of 8 group sessions and the participants could give feedback. In the second part, an HCI expert presented a walk-through of the companion app, and participants could give feedback.	Review of the intervention, both the group sessions and the companion app. Revision of the intervention, including limiting the amount of text and clarifying instructions in companion app.
Consultations with software company	We conducted several consultations with the software company that had the technical infrastructure for the companion app.	A fully functioning version of the companion app was completed.

^a^ADHD: attention-deficit/hyperactivity disorder.

^b^DBT: dialectic behavioral therapy.

^c^HCI: human-computer interaction.

^d^UX: user experience.

#### The Emotion Regulation Intervention for ADHD

##### Overview

The development process led to the “Emotion Regulation Intervention for ADHD” (ERIA), which is a structured psychological intervention aimed at improving emotion regulation skills in adults with ADHD. The intervention is manual-based and includes components from dialectic behavioral therapy skills training and positive psychology. ERIA consists of 8 face-to-face group sessions and a digital companion app for skills training in between sessions. Table 2 contains an overview of the intervention content in ERIA.

**Table 2:** Overview of the intervention, including session themes, content for group sessions, weekly homework, and skills training assignments.

Session	Content	Homework and skills training
1. Introduction	Introduction to group members and therapists Overview of the program ADHD^a^ a and emotion Discussion on challenges and strengths of ADHD Strategies for homework completion	Goal setting Identifying own strengths Complete plan for homework
2. Mindfulness I	Mindfulness practice Homework discussion Mindfulness Introduction to “what skills” in mindfulness Exercise: “observe object in room” Exercise: “describe the pictures”	Practice “what” skills in mindfulness (observation, description, and participation) Skills training log
3. Mindfulness II	Mindfulness practice Homework discussion Common barriers for homework completion Introduction to “how skills” in mindfulness Exercise: “judgmental vs nonjudgmental claims” Exercise: “multitasking”	Practice “how” skills in mindfulness (being nonjudgmental, doing 1 thing at the time, and doing what works) Skills training log
4. Emotion regulation I	Mindfulness practice Homework discussion Emotions and emotion regulation Emotion regulation skills: part I Introduction to the emotion diary Exercise: “linking situations to emotions”	Practice emotion regulation skills (observation of emotions and naming and describe emotions) Emotion diary Skills training log
5. Emotion regulation II	Mindfulness practice Homework discussion Adaptive vs maladaptive regulation strategies Emotion regulation skills: part II	Practice emotion regulation skills (check the facts, opposite action, and problem-solving) Emotion diary Skills training log
6. Emotion regulation III	Mindfulness practice Homework discussion Emotion regulation skills: part III Tips for planning and organization	Practice emotion regulation skills (covering basics needs and planning for challenging situations) Planning positive activities Emotion diary Skills training log
7. Crisis management	Mindfulness practice Homework discussion Skills for crisis management and intense emotions Discussion on distraction strategies	Practice skills in crisis management (stop and check-in, physical exercise, cold water, muscle relaxation, breath work, and distraction). Skills training log
8. Summary	Mindfulness practice Homework discussion Self-compassion Exercise: “supportive words” Summary of program Road ahead: maintaining change and setbacks	N/A^b^

^a^ADHD: attention-deficit/hyperactivity disorder.

^b^N/A: not applicable.

##### Group Sessions

ERIA comprises 8 weekly group sessions. The group sessions are closed, and each group includes 6-8 adults with ADHD. In this study, the groups were led by 2 clinical psychologists with a minimum of 3 years of clinical experience. Each group session lasts approximately 1.5 hours, divided into 2 segments with a 15-minute break interlude. All sessions begin with a brief mindfulness exercise, led by 1 of the 2 psychologists. Afterward, group members are encouraged to share their reflections on the exercise. Following this, the group members share their experiences with the previous week’s homework assignment. After the break, the lead psychologist presents relevant theoretical and psychoeducational information and the new skills for the participants to practice at home. There are also discussion breaks and some practical exercises incorporated in the presentation of new skills.

##### Companion App

The participants are given access to a companion app, which they are asked to use for skills training at home between the group sessions. This app is organized with modules, where new skills training modules are released on a weekly basis following the course of the group sessions. The skills training modules include the weekly skills that the participants are to practice at home. In addition, the participants can use the companion app to message the group leader, log their skills training sessions, and access the PowerPoint presentations for the group sessions, as well as other relevant resources (eg, relevant websites or scientific papers on the weekly theme). The participants also receive an SMS text message reminder when the skills training modules are available and another SMS text message reminder if they have not accessed the module within 2 days. Given that this was a feasibility study, the app did not encompass all features originally present in its prototype. For instance, a peer-support feature suggested during the co-design workshop was excluded due to its resource-intensive nature. If the feasibility study yields encouraging outcomes, we intend to incorporate more sophisticated features in the app.

The app was a web-based application that could be accessed both through a computer and a smartphone. To access the app, the participants had to use the Norwegian web-based authentication platform, BankID, for a secure 2-factor log-in. BankID is a widely used platform for electronic identification for services within banking, health care, and education in Norway. The companion app was hosted on the eHealth platform developed by the software company “Youwell,” which is partnered with the Western Norway Regional Health Authority for clinical use and research. The platform has a patient and therapist portal, where participants can access the app through the patient portal, and the therapist can set up the program and monitor their patient’s progress in the therapist portal. The platform is also used for building the app and allows for the input of content, such as text, audio, videos, and images, to create modules. It has previously been used for other internet-delivered programs, including programs for ADHD [36], social anxiety [37], and cognitive residual symptoms after depression [38].

**Figure 1:**
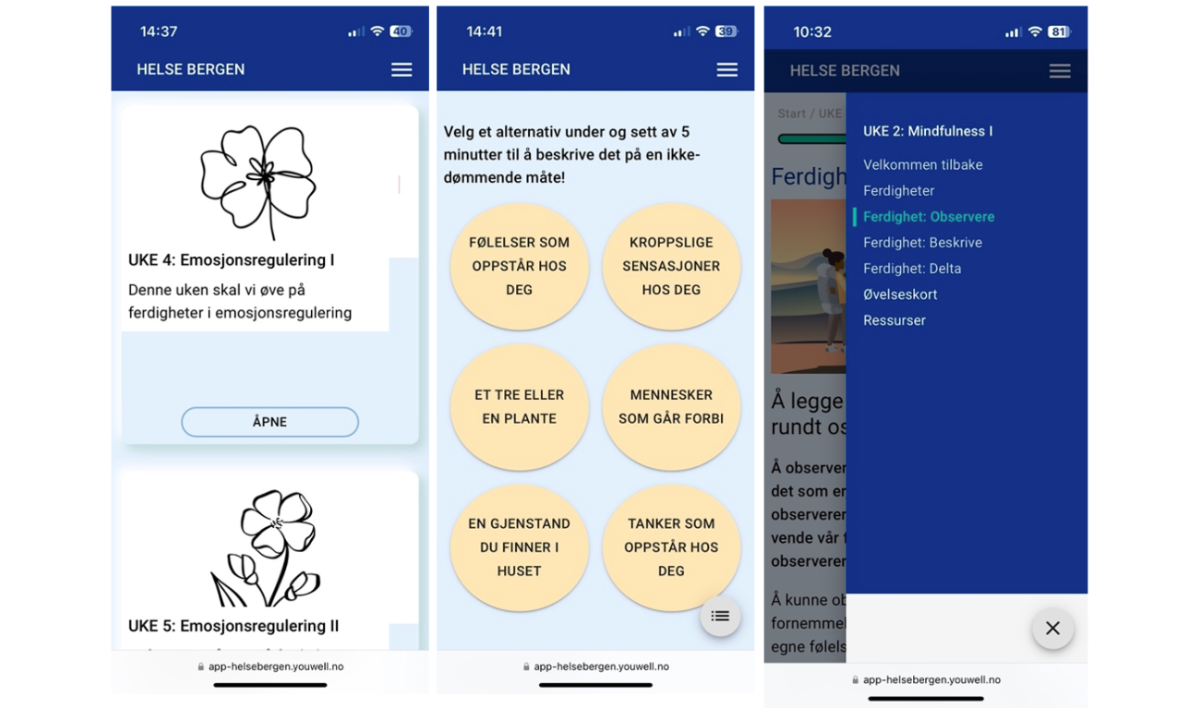
Overview of the companion app.

Figure 1 shows screenshots of the companion app. The first screen from the left shows the main page with an overview of the modules. The first box from the left reads, “Week 4: Emotion regulation I. This week we will practice skills in emotion regulation.” The second screen shows the instructions to a mindfulness skill with the text “Choose an option below and take 5 minutes to describe this in a nonjudgmental way.” The circles show 6 options they can choose from, which include describing (1) own emotions, (2) bodily sensations, (3) a tree or plant, (4) people walking by, (5) an object in the room, or (6) own thoughts. The third screen shows the side menu that participants can use to navigate within the module. This screen shows the mindfulness module and the side menu includes the specific module pages: “Welcome back,” “Skills,” “Observation,” “Description,” “Participate,” “Skills diary card,” and “Resources.”

### Phase 2: Feasibility Study

#### Participants

Eligible participants for this feasibility study were adults with ADHD living in Bergen, Norway. The inclusion criteria for the study were as follows: (1) minimum age of 18 years; (2) a diagnosis of ADHD according to the *DSM-5* (*Diagnostic and Statistical Manual of Mental Disorders* [Fifth Edition]) criteria; (3) current problems with emotion dysregulation as indicated by a score of ≥80 on the Difficulties in Emotion Regulation Scale (DERS); (4) having a smartphone or computer to access the companion app; and (5) the ability to attend in-person group sessions in Bergen, Norway. Exclusion criteria were (1) a high risk of suicidality, as indicated by having attempted suicide within the past year, having previously attempted suicide and reporting current suicidal ideations, or reporting current suicidal ideations and having a plan and preferred method; (2) co-occurring severe mental illness, including substance abuse disorder, psychosis, and major depressive disorder; and (3) current participation in another psychological treatment intervention. However, participants could still partake in the study if they had less severe psychiatric conditions, such as mild to moderate anxiety or depression, and if they received pharmacological treatment for their ADHD or other conditions.

#### Recruitment

Participants were recruited through the ADHD patient advocacy group “ADHD Norge,” which shared information about the study with its members through email and social media. The study opened for participants to sign up on November 24, 2022, and closed within a week (November 30, 2022) due to a large number of individuals signing up for the study. Interested participants signed up through a website that contained a screening survey to examine eligibility as well as information about the study. Eligible participants were contacted for a face-to-face screening with a clinical psychologist. During this screening, eligibility in terms of psychiatric comorbidities and suicidality was examined using the Mini International Neuropsychiatric Interview [39]. Participants were also asked about the date, clinic, and diagnosing clinician for their ADHD diagnosis. In addition, the participants were asked open questions about ADHD symptoms (ie, could you tell me about your current ADHD symptoms?) and everyday functioning (ie, how do you experience that ADHD affects you in your daily life?). The participants had to report symptoms and functional impairments that were in accordance with the DSM-5 criteria for ADHD, as assessed by a clinical psychologist. Those who were deemed eligible for participation following this screening were invited to take part in the study and to sign an informed consent form through the companion app.

#### Outcome Measures

##### Overview

The participants were given the preassessment 1 week before the intervention started and the postassessment 1 week after the intervention ended. The assessments were completed on the internet, except for the Behavior Rating Inventory of Executive Functioning–Adult version (BRIEF-A), which was given in-person at the first and last group session.

##### Credibility

The third item of the Credibility and Expectancy Scale (CEQ) was used to examine treatment credibility of the intervention [40]. The item states, “Would you recommend this treatment to a friend with similar challenges?” and the responses are given on a scale from 1 (not certain at all) to 9 (very certain).

##### Treatment Satisfaction

The participants were asked about whether they thought they would continue using the coping skills they had learned in the future and responded on a 4-point scale from 1 (not very likely) to 4 (very likely). They were also asked about which elements of the intervention they considered to be most useful (multiple choice with option to add own text). The participants were also asked about negative experiences with the intervention (yes or no), which they could elaborate on in an open-text field.

##### Adherence

Adherence was assessed by the number of group sessions attended and the number of completed skills training modules in the companion app. Participants that attended at least 6 of 8 group sessions were defined as treatment completers. The participants were also asked about how many days per week they practiced the coping skills.

##### Difficulties in Emotion Regulation Scale

DERS is a self-report questionnaire that is commonly used to assess emotion dysregulation in clinical populations [41]. The scale includes 36 items rated on a 5-point Likert scale ranging from 1 (never) to 5 (almost always), yielding a total score between 36 and 180, with 180 indicating the most severe problems with emotion dysregulation.

##### The Adult ADHD Self-Rating Scale

The Adult ADHD Self-Rating Scale (ASRS) is a self-report questionnaire that is used to assess symptoms of inattention and hyperactivity-impulsivity [42]. The scale includes 18 items, with 9 items reflecting inattentive symptoms and 9 items reflecting hyperactive-impulsive symptoms. Responses are given on a 5-point scale with options 0 (never), 1 (rarely), 2 (sometimes), 3 (often), or 4 (very often), giving a total score between 0 and 72 and a score between 0 and 36 for the inattention and hyperactivity-impulsivity subscales.

##### The Adult ADHD Quality of Life Measure

The Adult ADHD Quality of Life (AAQoL) measure is used to assess quality of life among adults with ADHD [43]. The scale includes 29 items rated on a scale from 1 (not at all or never) to 5 (extremely or very often), yielding a total score between 0 and 100.

##### Hospital Anxiety and Depression Scale

The Hospital Anxiety and Depression Scale (HADS) is a self-report questionnaire used to assess symptoms of depression and anxiety [44]. The scale includes 14 items, with 7 items reflecting anxiety symptoms and 7 items reflecting depressive symptoms. The response options range from 0 to 3, with 3 being the most severe level. The scale yields a total score between 0 and 42 and a score between 0 and 21 for the anxiety and depression subscales.

##### The Behavior Rating Inventory of Executive Functioning

BRIEF-A is a self-report questionnaire used to assess executive functioning in everyday life [45]. The scale consists of 75 items, which are rated on a 3-point scale (1=never, 2=sometimes, and 3=often). For this study, we report the Global Executive Composite score, which is an overall summary score including 9 clinical BRIEF-A subscales.

#### Statistical Analysis

SPSS software (IBM Corp) was used for all statistical analyses [46]. The participant demographics, adherence measures, and treatment credibility measures were assessed using descriptive statistics, which include calculation of means, frequencies, ranges, and SDs. To evaluate preliminary clinical outcomes, paired sample *t* tests were used with an initial significance level set at .05. Due to the risk of family-wise error (type I error) associated with multiple *t* tests, a Bonferroni correction was included. This adjustment was achieved by dividing the α level by the number of conducted hypotheses tests, that is, *t* tests (.05/8), resulting in a corrected significance level of .006. The choice of analytic approach necessitated the inclusion of cases with both pre- and postassessment. The magnitude of treatment effect was quantified using standardized effect sizes, estimated through Cohen *d*, with the formula (M_2_ – M_1_)⁄SD_pooled_. The pooled SD was calculated by √([SD_1_^2^ + SD_2_^2^]⁄2). Effect sizes were interpreted according to conventions, where *d=*0.20, *d=*0.50, and *d*≥0.80 are defined as small, moderate, and large, respectively.

### Ethical Considerations

The study was reviewed and approved by the Regional Ethics Committee of Norway, Region West (494659). The participants were informed about the study and their rights both in-person and in writing. Before participation, all participants signed an informed consent form, acknowledging that they could withdraw their consent at any time without any repercussions. The data were pseudoanonymized and stored on a dedicated research server according to regulative standards at the university and hospital. The participants were compensated NOK 1000 (US $90) for their participation in the study.

## Results

### Participants

A total of 16 participants took part in this study (Table 3 shows participant characteristics). The majority were diagnosed with ADHD in adulthood, with the mean age for receiving the diagnosis being 30.4 (SD 11.7) years.

**Figure 2:**
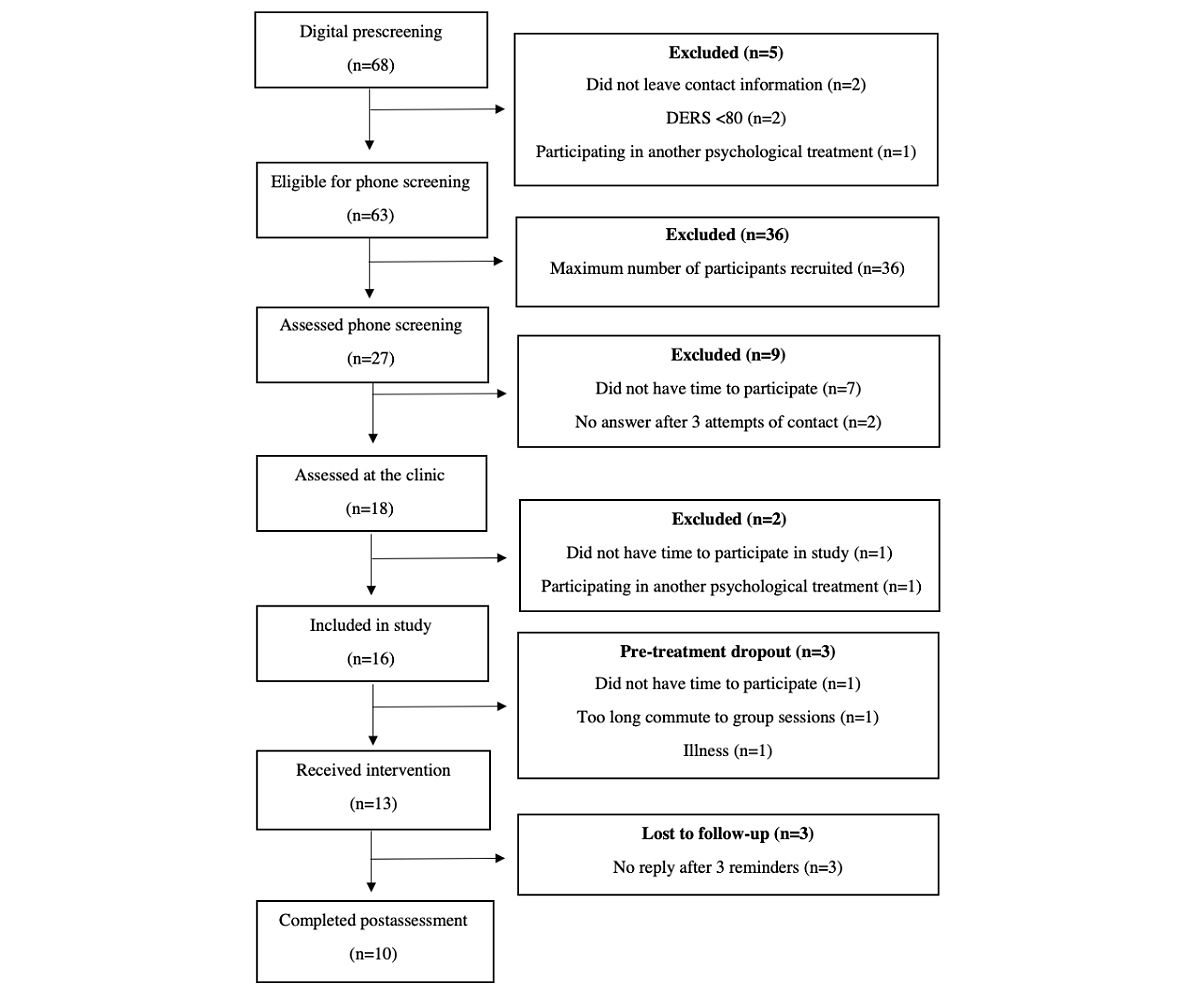
Study flowchart. DERS: Difficulties in Emotion Regulation Scale.

All participants were recruited within 1 week. During the recruitment period, 68 adults completed the digital prescreening, of whom 92.6% (n=63) met the inclusion criteria and provided contact information (Figure 2). However, due to the limited number of study places in this feasibility study, we only assessed the first 27 individuals who signed up for the phone screening, as this was sufficient to reach the maximum capacity of 16 included participants. We sent an SMS text message to the remainder of the people who signed up, informing them that the study was fully booked. To ensure a more balanced sex distribution in the study, 6 study places were reserved for men, as we wanted to make sure we received feedback from both sexes.

**Table 3:** Participant characteristics in the feasibility study (n=16).

Characteristic		Values, n (%)
**Sex**		
	Female	10 (62.5)
	Male	6 (37.5)
**Age group (years)**		
	18-34	7 (43.8)
	35-44	7 (43.8)
	45-55	1 (6.2)
	55-64	1 (6.2)
**Educational level**		
	High school	8 (50)
	College or university level	8 (50)
**Occupational status**		
	Employed or student	9 (56.2)
	Work assessment allowance or sick leave	6 (37.5)
	Disability pension	1 (6.2)
**ADHD medication status**		
	Daily medication usage	13 (81.3)
	Weekly or monthly usage	2 (12.4)
	Rarely, a few times a year	1 (6.2)

### Treatment Adherence, Credibility, and Satisfaction

In terms of adherence, a total of 3 participants dropped out of the study before starting the intervention, making a pretreatment dropout rate of 18.8% (3/16). Among the participants who received the intervention (n=13), the average number of group sessions attended was 6.2 out of 8 sessions. A total of 4 (26.6%) participants were treatment dropouts, defined as participants who attended less than 6 group sessions. Consequently, the cumulative dropout rate, including the participants who dropped out before starting the intervention, reached 43.7% (n=7). Regarding the use of the companion app, the participants had a mean completion of 6.8 out of 8 modules. On average, the participants reported practicing the skills 4.3 days per week.

The intervention was generally well received in terms of treatment credibility with a mean rating of 7.1 (SD 2.6; range 1-9). Among the 10 participants who completed the postassessment, 7 participants reported that they were very certain or certain that they would recommend the treatment to a friend facing similar challenges as themselves; 2 were somewhat certain, whereas 1 was not at all certain about recommending it.

Feedback from participants highlighted overall satisfaction with the intervention, with a mean rating of 3.3 (SD 0.9; range 1-4). All but 1 participant planned to continue using the learned skills. The participants rated meeting others with ADHD (n=10), the in-person group sessions (n=7), the skills (n=7), and therapist support (n=4) as the most useful elements of the intervention. Their feedback also included suggestions for improvement, with 3 participants recommending more time for group interactions and discussions among the group members. Regarding this, 1 participant suggested that the group members should be able to interact between the sessions through the companion app. Other suggestions for improvements included an extension of the program by adding more group sessions, making the companion app available for direct download, incorporating more reminders, providing participants with a printed version of the skills, and involving an individual with ADHD as a group presenter for skills demonstration and experience sharing. While feedback was largely positive, 2 participants expressed negative experiences with the program: 1 found the skills in the crisis management module to be inadequate in emotional crises, and another felt that the intervention was not sufficiently tailored to ADHD. Importantly, there were no reports of clinical deterioration among the participants.

### Clinical Outcomes

There was an overall statistically significant decrease in self-reported emotion dysregulation from before to after treatment, with a strong effect size (*d*=2.0). Table 4 shows the individual scores for emotion dysregulation, indicating a change in the positive direction for all participants.

Overall, the group of participants showed a significant decrease in secondary clinical outcome scores, reflecting the level of inattention and hyperactivity-impulsivity from before to after assessment. While initial analyses showed a change in quality of life and depressive symptoms, these changes were nonsignificant with a Bonferroni correction. No significant improvement was found in measures of executive functioning or anxiety symptoms (Table 5 contains an overview of all preliminary clinical outcomes).

**Table 4:** Individual scores for the Difficulties in Emotion Regulation Scale from before to after assessment (n=10).

Participant	Preassessment scores	Postassessment scores
1	130	101
2	138	84
3	110	93
4	147	91
5	96	83
6	130	94
7	107	96
8	139	117
9	133	95
10	107	100

**Table 5:** Clinical outcomes of emotion dysregulation, inattention, hyperactivity-impulsivity, quality of life, depression, anxiety, and executive functioning from before to after assessment (n=10).

Outcome measure	Preassessment, mean (SD)	Postassessment, mean (SD)	*P* value^a^	Effect size, Cohen *d*	Difference, 95% CI
DERS^b^	123.7 (17.2)	95.4 (9.6)	<.001	2.0	15.8 to 40.8
ASRS^c^ full scale	51.9 (9.6)	41.9 (8.9)	<.001	1.1	6.2 to 13.8
ASRS inattention	26.9 (4.1)	22.3 (5.2)	<.001	1.0	2.3 to 6.9
ASRS hyperactivity-impulsivity	25.0 (6.4)	19.6 (5.1)	.002	0.9	2.5 to 8.3
AAQoL^d^	44.4 (9.6)	56.7 (11.8)	.03	—^e^	–23.3 to –1.3
HADS^f^ Anxiety	10.8 (4.3)	9.8 (3.6)	.24	—	–0.8 to 2.8
HADS Depression	6.0 (3.7)	4.3 (3.2)	.03	—	0.2 to 3.2
BRIEF-A^g^ GEC^h^	154.4 (15.9)	150.8 (12.6)	.20	—	–7.8 to 15.1

^a^Significance level set to .006 with Bonferroni correction.

^b^DERS: Difficulties in Emotion Regulation Scale.

^c^ASRS: Adult ADHD Self-Rating Scale.

^d^AAQoL: Adult ADHD Quality of Life.

^e^Not available.

^f^HADS: Hospital Anxiety and Depression Scale.

^g^BRIEF-A: Behavior Rating Inventory of Executive Functioning.

^h^GEC: Global Executive Composite.

## Discussion

### Principal Findings

The aim of this study was to describe the development and assess the feasibility of ERIA, a blended digital and face-to-face intervention targeting emotion dysregulation in adults with ADHD. Overall, the findings were promising and supported the feasibility of ERIA. Both treatment satisfaction and credibility were generally good, which aligned with findings from other psychological interventions targeting emotion dysregulation in adults with ADHD [26]. More specifically, the participants emphasized the group component and meeting others with ADHD as useful aspects of the intervention. This is in line with previous studies showing that providing a forum where one can share experiences with peers is particularly valuable in treatment settings for this group of adults [47]. While the results are promising, there is room for refinements, including an even better tailoring of the intervention to ADHD. An important step forward would be to involve adults with ADHD in the review and refinement process to ensure that all key aspects of the interventions are well-adapted to the challenges and difficulties facing adults with ADHD. Nonetheless, the heterogeneous nature of ADHD requires finding a balance between including intervention components that fit most participants and including specialized strategies addressing the specific needs of a subset of individuals with ADHD.

This study highlighted concerns regarding treatment adherence, especially regarding pretreatment dropout. A total of 3 participants dropped out before starting the intervention, while another 4 participants did not complete the intervention. This finding aligns with well-known challenges related to treatment adherence among adults with ADHD [48,49]. The issues related to adherence in this study may be partially attributed to our community-based sample. Motivation for completing psychological treatment may thus have been lower than among clinic-recruited participants. However, there could also be intervention-specific factors related to dropouts, such as dissatisfaction with the intervention or finding the intervention too demanding. Yet it is also worth noting that although high levels of treatment adherence are generally seen as favorable for the patient, treatment dropout does not necessarily equate to clinical failure for the patients themselves. Still, when reviewing the current intervention, it may be necessary to include more strategies to prevent early termination of treatment. A literature review on the topic found that strategies such as pretherapy preparation, patient selection, time-limited treatment contracts, appointment reminders, motivation enhancement, facilitation of a therapeutic alliance, and facilitation of affect expression were specific strategies that could be applied to reduce premature termination of treatment across different psychiatric disorders [50]. As we progress, understanding the multifaceted factors associated with dropout will be crucial.

With regard to the companion app, it is interesting to note that the participants who initiated the intervention generally completed a high number of skills training modules in the companion app, with the majority completing all modules. This finding is in line with the results from Selaskowski et al [35], which showed that the inclusion of a mobile app in a group-based intervention for adults with ADHD was linked to higher homework compliance [35]. Given the importance of homework for behavioral change as well as the common challenges related to homework compliance in psychological interventions, facilitating methods to ensure high attrition of homework may be particularly useful [51]. However, it is important to note that with the design of this study, we cannot determine whether the companion app resulted in higher homework compliance as opposed to not including the app; this would be an interesting topic for future studies.

The results were promising in terms of preliminary clinical findings, with participants showing a significant and large reduction from pre- to post treatment in emotion dysregulation. These findings are in accordance with findings from other psychological interventions targeting emotion dysregulation in individuals with ADHD [26]. Significant improvements were also observed in the secondary clinical outcome measures, including inattention and hyperactivity-impulsivity. However, given the study’s small scale, nonrandomized, and uncontrolled design, the clinical findings must be considered with caution. Moreover, we found no significant changes in the measures of anxiety, depression, quality of life, or executive functioning. Regarding executive functioning, previous research has shown that measures of executive control remain stable in ADHD, regardless of remission or persistence [52]. More generally, the effect of interventions that are designed to improve cognitive abilities or executive functioning appears to be domain-specific and show mixed results [53,54].

Taken together, the results from this study were encouraging and call for further development of the intervention and a more extensive examination of clinical effects in a randomized controlled trial.

### Limitations

This feasibility study has some limitations that should be noted, in particular the small sample size, the absence of a control group, and the lack of randomization. Due to these limitations, conclusions regarding the intervention’s clinical effects remain elusive, and we cannot rule out placebo or other random effects. Yet, the aim of this study was not to examine the effectiveness of the intervention but rather to assess its feasibility before paving the way for larger trials.

A further limitation is that 3 participants who took part in the intervention did not complete the postassessment. Therefore, it is possible that this could have impacted the treatment satisfaction and credibility scores of the interventions. Furthermore, the participants were recruited from the community and may therefore differ in some way from typical clinic-recruited adults with ADHD. The reliance on self-report scales to evaluate clinical outcomes in this study should also be considered a limitation. In future studies, it would be interesting to include other measures, such as those generated from sensor technology, clinician ratings, and performance on cognitive tests.

### Conclusion

In conclusion, the results from this feasibility study support the potential of ERIA as a feasible intervention for addressing emotion dysregulation in adults with ADHD and call for further investigation in a randomized controlled trial. The blended approach, integrating digital and face-to-face elements, may offer some advantages compared to an exclusively digital or face-to-face treatment format. The in-person group sessions were especially valued because they provided opportunities to interact with peers. Meanwhile, the high completion rate of the companion app modules indicates their potential to facilitate skills training.

## Data Availability

The data sets generated and analyzed during this study are available from the corresponding author on reasonable request.

## Abbreviations

AAQoL: Adult ADHD Quality of Life
ADHD: attention-deficit/hyperactivity disorder
ASRS: Adult ADHD Self-Rating Scale
BRIEF-A: Behavior Rating Inventory of Executive Functioning–Adult version
CEQ: Credibility and Expectancy Scale
DERS: Difficulties in Emotion Regulation Scale
DSM-5: Diagnostic and Statistical Manual of Mental Disorders (Fifth Edition)
ERIA: Emotion Regulation Intervention for ADHD
GEARS: Group Therapy for Improving Emotional Acceptance and Regulatory Skills in Adults with ADHD
HADS: Hospital Anxiety and Depression Scale
